# Poly[[(μ_4_-benzene-1,3,5-tri­carboxyl­ato-κ^4^ *O* ^1^:*O* ^1′^:*O* ^2^:*O* ^3^)bis­(2,2-bi­pyridine-κ^2^ *N*,*N*′)(μ_2_-hydroxido)dicopper(II)] trihydrate]

**DOI:** 10.1107/S1600536814013877

**Published:** 2014-06-21

**Authors:** Mohamed N. El-kaheli, Ramadan M. El-mehdawi, Ramadan G. Abuhmaiera, Mufida M. Ben Younes, Fathia A. Treish, Annalisa Guerri, Carla Bazzicalupi

**Affiliations:** a Chemistry Department, Tripoli University, Tripoli, Libya; b Chemistry Department "U. Schiff", University of Florence, Florence, Italy

**Keywords:** Copper nitrate, benzene-1,3,5-tri­carb­oxy­lic acid, Bi­pyridine, Topology, Two-dimension., crystal structure

## Abstract

In the title two-dimensional coordination polymer, {[Cu_2_(C_9_H_3_O_6_)(OH)(C_10_H_8_N_2_)_2_]·3H_2_O}_*n*_, each of the two independent Cu^II^ atoms is coordinated by a bridging OH group, two O atoms from two benzene-1,3,5-tri­carboxyl­ate (*L*) ligands and two N atoms from a 2,2- bi­pyridine (bipy) ligand in a distorted square-pyramidal geometry. Each *L* ligand coordinates four Cu^II^ atoms, thus forming a polymeric layer parallel to the *bc* plane with bipy molecules protruding up and down. The lattice water mol­ecules involved in O—H⋯· O hydrogen bonding are situated in the inner part of each layer. The crystal packing is consolidated by π–π inter­actions between the aromatic rings of bipy ligands from neigbouring layers [inter­centroid distance = 3.762 (3) Å].

## Related literature   

For general background, see: Napolitano *et al.* (2008 ▶). For a coordination polymer containing benzene­tricarboxlyate, see: Wang *et al.* (2005 ▶). For Cu—O bond-length data, see: Janiak *et al.* (2008 ▶); Rogan *et al.* (2011 ▶). For related structures, see: Christou *et al.* (1990 ▶); Tokii *et al.* (1992 ▶).
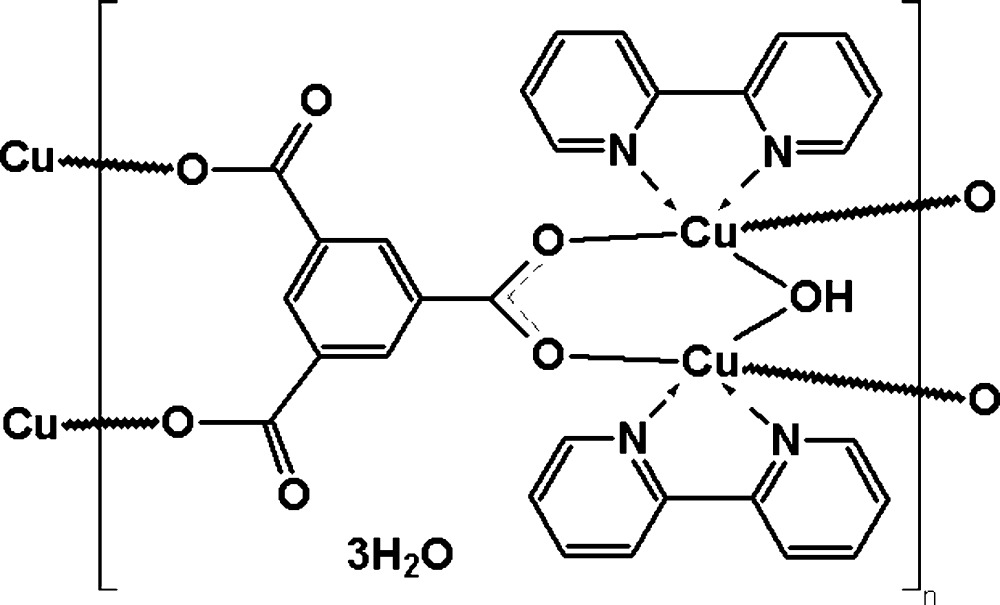


## Experimental   

### 

#### Crystal data  

[Cu_2_(C_9_H_3_O_6_)(OH)(C_10_H_8_N_2_)_2_]·3H_2_O
*M* *_r_* = 717.62
Monoclinic, 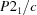

*a* = 16.493 (1) Å
*b* = 9.7017 (5) Å
*c* = 17.908 (1) Å
β = 102.426 (6)°
*V* = 2798.1 (3) Å^3^
*Z* = 4
Mo *K*α radiation
μ = 1.59 mm^−1^
*T* = 150 K
0.2 × 0.2 × 0.1 mm

#### Data collection  

Oxford Diffraction Xcalibur Sapphire3 diffractometer
Absorption correction: multi-scan (*CrysAlis PRO*; Oxford Diffraction, 2010 ▶) *T*_min_ = 0.760, *T*_max_ = 0.810
11267 measured reflections
6245 independent reflections
4210 reflections with *I* > 2σ(*I*)
*R*_int_ = 0.035

#### Refinement  

*R*[*F* ^2^ > 2σ(*F* ^2^)] = 0.042
*wR*(*F* ^2^) = 0.099
*S* = 0.94
6245 reflections
434 parameters
1 restraint
H atoms treated by a mixture of independent and constrained refinement
Δρ_max_ = 1.45 e Å^−3^
Δρ_min_ = −0.66 e Å^−3^

### 

Data collection: *CrysAlis CCD* (Oxford Diffraction, 2007 ▶); cell refinement: *CrysAlis RED* (Oxford Diffraction, 2007 ▶); data reduction: *CrysAlis RED*; program(s) used to solve structure: *SIR92* (Altomare *et al.*, 1993 ▶); program(s) used to refine structure: *SHELX2013* (Sheldrick, 2008 ▶); molecular graphics: *Mercury* (Macrae *et al.*, 2008 ▶); software used to prepare material for publication: *publCIF* (Westrip, 2010 ▶).

## Supplementary Material

Crystal structure: contains datablock(s) I, New_Global_Publ_Block. DOI: 10.1107/S1600536814013877/cv5448sup1.cif

Structure factors: contains datablock(s) I. DOI: 10.1107/S1600536814013877/cv5448Isup2.hkl

CCDC reference: 921256

Additional supporting information:  crystallographic information; 3D view; checkCIF report

## Figures and Tables

**Table 1:** Hydrogen-bond geometry (Å, °)

*D*—H⋯*A*	*D*—H	H⋯*A*	*D*⋯*A*	*D*—H⋯*A*
O*W*1—H1*W*1⋯O*W*3^i^	0.76 (4)	2.09 (4)	2.834 (5)	168 (4)
O*W*1—H2*W*1⋯O4	0.84 (5)	2.08 (5)	2.878 (4)	159 (4)
O*W*3—H1*W*3⋯O3	0.72 (5)	2.18 (5)	2.880 (4)	165 (6)
O*W*3—H2*W*3⋯O*W*2^ii^	0.89 (4)	1.88 (4)	2.729 (5)	161 (4)
O*W*2—H1*W*2⋯O5^i^	0.79 (4)	1.94 (5)	2.716 (5)	164 (4)
O*W*2—H2*W*2⋯O7	0.79 (6)	2.01 (6)	2.774 (6)	163 (6)
O2—H*O*2⋯O5^iii^	0.90 (2)	2.35 (3)	2.943 (3)	123 (3)
O2—H*O*2⋯O7^iv^	0.90 (2)	2.37 (4)	2.884 (3)	116 (3)

Symmetry codes: (i) 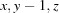; (ii) 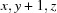; (iii) 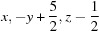; (iv) 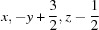.

**Fig. 1.**
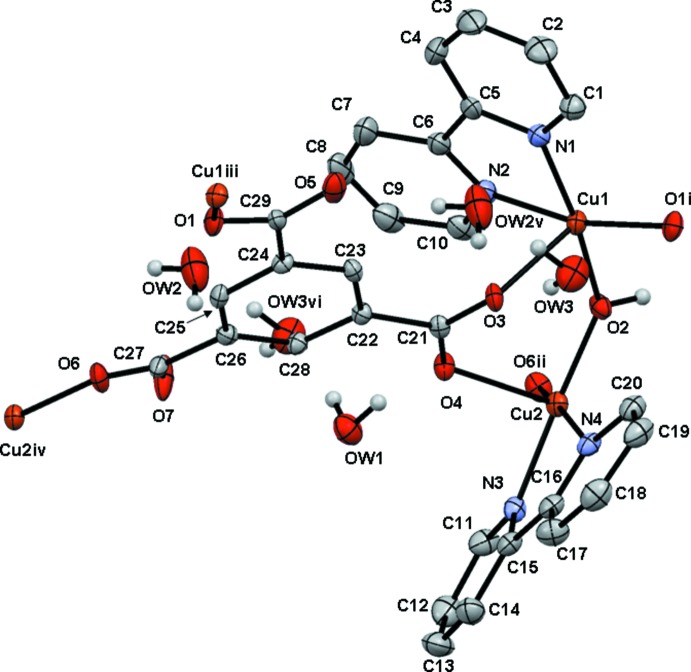
A portion of (1), showing the atom numbering scheme. Displacement ellipsoids are drawn at the 50% probability level. C-bound H atoms omitted for clarity. [Symmetry codes: (i) x, -y + 5/2, z-1/2; (ii) x, -y + 3/2, z - 1/2; (iii) x, -y + 5/2, z+1/2; (iv) x, -y + 3/2, z+1/2; (v) x, y + 1, z; (vi) x, y - 1, z].

**Fig. 2.**
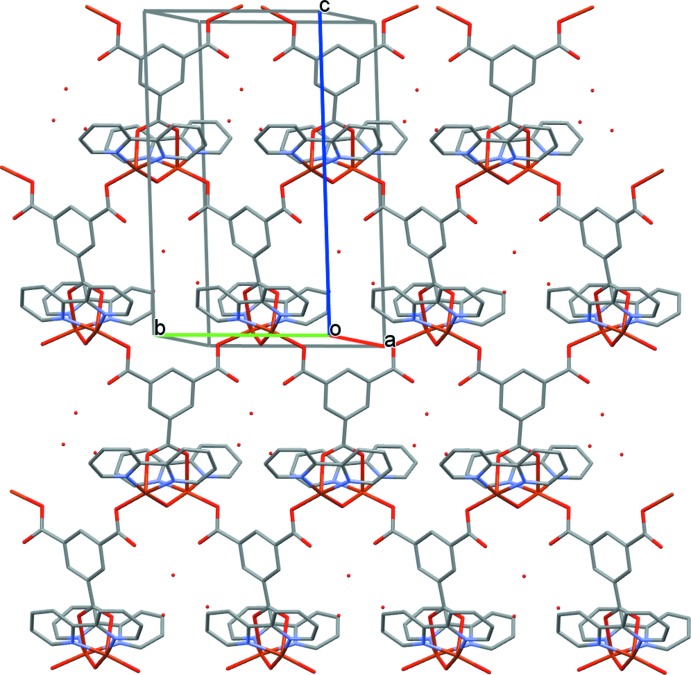
A portion of the crystal packing showing two-dimensional undulated layer parallel to the [10–1] plane.

**Fig. 3.**
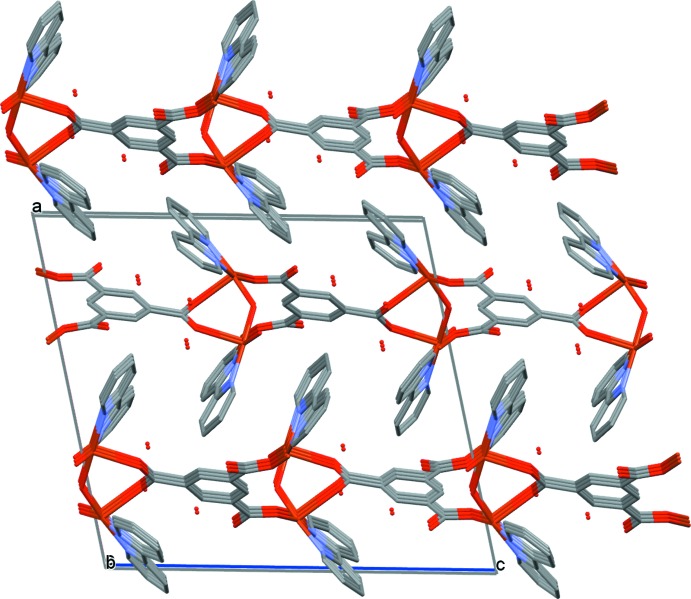
A portion of the crystal packing viewed approximately down the b axis.

## supplementary crystallographic information

### S1. Introduction

The design and synthesis of metal-organic framework has been an area of rapid growth in recent years owing to the potential application and as zeolite-like material for molecular selection (Napolitano *et al.*,2008). Polycarboxyl­ate ligands present very rich coordination chemistry, because of their ability to bridge transition metal ions generating various polynuclear complexes. Aromatic polycarboxyl­ate are of high inter­est due to their versatility in constructing coordination complexes, and 1,3,5-benzene tri­carb­oxy­lic acid have been proved to be efficacious towards preparation of metal-organic coordination complexes. Moreover, these carboxyl­ate bridges provide a means for efficiently transmitting magnetic information. During the last decade, many reports appeared on the synthesis of coordination compounds where trianions of benzene-1,3,5-tri­carb­oxy­lic acid combined with aromatic N-containing chelating ligands have been used to essemble a wide range of coordination polymers from chains, to networks (Wang *et al.*,2005). Usually the construction of molecular architecture depends on several factors such as coordination geometry of metal ions, organic ligands, counter ions, solvents and temperature. Due to the flexible nature of Cu^II^ coordination sphere, assisted by the Jahn-Teller effect which can be realized either by distortion of an o­cta­hedral geometry to give a 4 +1+1 bonding, or else by a change in coordination number as an alternative means of lifting the degeneracy of unequally occupied d-orbitals so copper will be the best choice. Herein, we report the synthesis and crystal structure of a new two-dimensional Cu^II^ complex-coordination polymer containing aromatic polycarb­oxy­lic ligand such as benzene-1,3,5-tri­carb­oxy­lic acid and hetero aromatic ligand such as 2,2-bi­pyridine. Our inter­est in dimeric bifunctional materials is direct toward the effects of weak inter­actions between molecular units, since the stacking of bipy rings is a potential source of inter­molecular exchange couplings.

### S2. Experimental

All starting materials were commercial products and were used as supplied from the Aldrich Company.

### S2.1. Synthesis and crystallization

The title complex was prepared by refluxing 1,3,5-benzene­tri­carb­oxy­lic acid (0.25 m mol, 0.05g) and 2,2—bi­pyridine ( 0.5 m mol, 0.078 g ) with Cu(NO_3_)_2_.3H_2_O ( 1.0 m mol, 0.241 g ) in 20% ethano­lic solution in the presence of NaOH (2.0 m mol, 0.08 g). Prismatic blue crystals suitable for X-ray analysis were obtained within one week by slow evaporation of an ethanol solution.

### S2.2. Refinement

C-bound H atoms were geometrically positioned and refined as riding. The O-bound H atoms were located on the Fourier difference map and isotropically refined. For the hydroxo group, the O—H bond distance has been restrained to 0.90 (2) Å.

### S3. Results and discussion

In the title complex (I), the dinuclear copper (II) coordination polymer (Fig. 1), features two very similar pyramidal CuN_2_O_3_ chromophores both adopting a (4+1) slightly distorted square-pyramidal arrangement, which share one vertex occupied by a bridging hydroxide group. The hydroxide group occupies one of the basal positions of both the CuN_2_O_3_ square pyramids, so that the inter­metallic distance is 3.5251 (6)Å. The oxygen atoms of a syn-anti triatomic carboxyl­ate bridge occupy the apical positions of the two coordination spheres. These Cu—O distances are very close to those reported for [Cu_2_(µ_5_-btb)(µ-OH)(µ-H_2_O)]_n_ (btb= benzene-1,2,3-tri­carboxyl­ate) (Janiak *et al.*, 2008) and shorter than that reported for {[Cu(C_8_H_4_O_4_)(C_10_H_9_N_3_)].H_2_O}_n_ (Rogan *et al.*, 2011). The rest of the basal sites of each Cu^II^ centre are occupied by a monodentate carboxyl­ate oxygen of another BTC^3-^ ligand, and completed by an N,N-chelating di­pyridine ligand. The shortest inter­chain separation of the metal centres is 9.7017 (7)Å , and 9.7348 (7)Å between the layers.

As expected for Cu^II^ in square–pyramidal geometry, the apical Cu—O bond distance is significantly longer than the remaining four distances in the Cu coordination polyhedron. This circumstance is characteristic of Jahn-Teller systems. Additional short Cu1—O5 and Cu2—O7 contacts, 2.935 (2) and 2.866 (2) Å respectively, are almost equal or slightly shorter than the sum of the van der Waals radii ( 2.92 Å), and also slightly shorter than 3.0229Å ( Rogan *et al.*, 2011). Since the O3—Cu1—O5, and O4—Cu2—O7 angles are 145.28 (8) and 143.84 (8) deg , respectively , the Cu1 and Cu2 enviroments could be described as an elongated o­cta­hedrons. The structure of the title complex with Cu····Cu separation of 3.5251 (6) consists of a doubly bridging pair of coordinate copper atoms, but only of the bridging ligand is carboxyl­ate group in its syn-anti mode, the other being an OH^-^ ion is conciderably longer compared with those seen in classic Cu_2_(O_2_CR)_4_L_2_ structures where the four bidentate bridging carboxyl­ates allow a much closer approach to the metals ( 2.6-2.7Å ). The Cu····Cu separation in complex (1) is short compared with that in [Cu_2_(btb)( µ-OH)(µ-H_2_O)]_n_ (Janiak *et al.*, 2008) coordination polymer which contains two crystallographically independent Cu^II^ atoms , bridged by a hydroxo ligand and a syn-syn coordinated carboxyl­ate group (Cu····Cu = 3.083 Å ) or by a syn-anti-coordinated carboxyl­ate group ( Cu····Cu = 5.447 Å ) . Each bi­pyridine ligand coordinates one metal ion occupying two adjacent basal coordination sites. As a consquence, both of them features convergent nitro­gen atoms and almost coplanar aromatic rings, the N—C—C—N torsion angles being -7.0 (4) and -0.9 (4) deg, for N1—C5—C6—N2 and N3—C15—C16—N4, respectively. The C5—C6 and C15—C16 bond lengths are as expected ( C5—C6 1.476 (5) Å and C15—C16 1.479 (5) Å .

The BTC^3-^ trianion acts as a tetra­dentate ligand with monodentate (C29/O5/O1) and (C27/O7/O8) for Cu1 and Cu2 respectively, and bridging (C21/O3/O4) carboxyl­ate groups featuring C—O bonds almost perfectly resonant [C21—O3 =1.263 (4)Å and C21—O4 = 1.261 (4)Å]. As a consequence each BTC^3-^ bridges three [dipy_2_Cu_2_(µ-OH)] units forming a two dimensional network growing perpendicularly to the *a* axis (Fig.2 ). This network can be described as a honeycomb structure (Fig. 2 ), formed by irregular hexagons sharing their edges and whose vertices are constituted by alternated tri­carboxyl­ate and bimetallic [dipy_2_Cu_2_(µ-OH)] units. The two-dimensional networks stack parallel to each other at an inter­planar distance of 8 Å. This inter­planar space is filled by the bi­pyridine moieties from the bimetallic units of two adjacent networks (Fig. 3 ). In particular, the bi­pyridine groups belonging to superposed bimetallic units, symmetry related by an inversion centre inter­act, inter­acts via face-to-face π-stacking: in each couple the two inter­acting pyridine rings are nearly parallel, with an inter­planar distance of 3.57 (3) Å and a ring centroid-ring centroid offset of 2.45 (3) Å. Additional carbon carbon contacts (3.529 (6) Å) connects bi­pyridine moieties symmetry related by screw axis. The inter­actions involving all the bi­pyridine groups above and below the honeycomb structure provide an overall strong connection along the third packing dimension, since the stacking of bi­pyridine rings is a potential source of weak inter­molecular exchange coupling.

Further analysis of the packing structure reveals that this structure contains three water molecules in the lattice which are localized inside the honeycomb hexagons. There are short inter­chain water-carboxyl­ate and water-water contacts that are indicative of a hydrogen bonding (Table 1). The hydrogen atom of the hydroxo bridge participate in classical O—H····O bonding with O5 of the carboxyl­ate group of another molecule (Table 1). The multidimensional framework structures formed by these combination of aromatic ligands are often stabilized *via* noncovalent inter­molecular forces, *viz*. hydrogen bonds and π–π inter­actions . In summary, benzene­polycarb­oxy­lic acids and N-containing chelating aromatic compounds have promoted the construction of multi-dimensional networks. Variation of the carb­oxy­lic acid elements along with the poly-N-chelating aromatic complexes is envisioned to produce materials, which could find potential application in self-assembled nanoscale molecular devices.

### Crystal data

**Table:**

[Cu_2_(C_9_H_3_O_6_)(OH)(C_10_H_8_N_2_)_2_]·3H_2_O	*F*(000) = 1456
*M**_r_* = 717.62	*D*_x_ = 1.703 Mg m^−^^3^
Monoclinic, *P*2_1_/*c*	Mo *K*α radiation, λ = 0.71073 Å
*a* = 16.493 (1) Å	Cell parameters from 4560 reflections
*b* = 9.7017 (5) Å	θ = 4.2–28.8°
*c* = 17.908 (1) Å	µ = 1.59 mm^−^^1^
β = 102.426 (6)°	*T* = 150 K
*V* = 2798.1 (3) Å^3^	Prismatic, blue
*Z* = 4	0.2 × 0.2 × 0.1 mm

### Data collection

**Table:**

Oxford Diffraction Xcalibur Sapphire3 diffractometer	6245 independent reflections
Radiation source: Enhance (Mo) X-ray Source	4210 reflections with *I* > 2σ(*I*)
Graphite monochromator	*R*_int_ = 0.035
Detector resolution: 16.4547 pixels mm^-1^	θ_max_ = 28.9°, θ_min_ = 2.3°
ω scan	*h* = −20→18
Absorption correction: multi-scan (*CrysAlis PRO*; Oxford Diffraction, 2010)	*k* = −10→12
*T*_min_ = 0.760, *T*_max_ = 0.810	*l* = −24→20
11267 measured reflections	

### Refinement

**Table:**

Refinement on *F*^2^	Primary atom site location: structure-invariant direct methods
Least-squares matrix: full	Secondary atom site location: difference Fourier map
*R*[*F*^2^ > 2σ(*F*^2^)] = 0.042	Hydrogen site location: mixed
*wR*(*F*^2^) = 0.099	H atoms treated by a mixture of independent and constrained refinement
*S* = 0.94	*w* = 1/[σ^2^(*F*_o_^2^) + (0.0493*P*)^2^] where *P* = (*F*_o_^2^ + 2*F*_c_^2^)/3
6245 reflections	(Δ/σ)_max_ = 0.001
434 parameters	Δρ_max_ = 1.45 e Å^−^^3^
1 restraint	Δρ_min_ = −0.66 e Å^−^^3^

### Special details

**Table:**

Experimental. Absorption correction: CrysAlisPro, Oxford Diffraction Ltd., Version 1.171.34.44 (release 25-10-2010 CrysAlis171 .NET) (compiled Oct 25 2010,18:11:34) Empirical absorption correction using spherical harmonics, implemented in SCALE3 ABSPACK scaling algorithm.
Geometry. All e.s.d.'s (except the e.s.d. in the dihedral angle between two l.s. planes) are estimated using the full covariance matrix. The cell e.s.d.'s are taken into account individually in the estimation of e.s.d.'s in distances, angles and torsion angles; correlations between e.s.d.'s in cell parameters are only used when they are defined by crystal symmetry. An approximate (isotropic) treatment of cell e.s.d.'s is used for estimating e.s.d.'s involving l.s. planes.

### Fractional atomic coordinates and isotropic or equivalent isotropic displacement parameters (Å^2^)

**Table:**

	*x*	*y*	*z*	*U*_iso_*/*U*_eq_	
Cu1	0.15663 (3)	1.12441 (4)	0.51735 (2)	0.01664 (11)	
Cu2	0.34511 (3)	0.94695 (4)	0.52697 (2)	0.01641 (11)	
N1	0.06719 (18)	1.2037 (3)	0.56556 (15)	0.0193 (6)	
N2	0.11195 (18)	0.9485 (3)	0.55432 (16)	0.0205 (6)	
N3	0.45778 (18)	0.8754 (3)	0.57948 (15)	0.0184 (6)	
N4	0.40277 (18)	1.1262 (3)	0.56267 (15)	0.0177 (6)	
O1	0.17136 (15)	1.2007 (2)	0.96699 (12)	0.0212 (6)	
O2	0.24185 (15)	1.0324 (2)	0.48006 (12)	0.0192 (5)	
O3	0.24286 (15)	1.1348 (2)	0.63702 (12)	0.0179 (5)	
O4	0.30490 (15)	0.9281 (2)	0.64187 (12)	0.0173 (5)	
O5	0.13890 (16)	1.3084 (2)	0.85481 (13)	0.0247 (6)	
O6	0.31266 (15)	0.7355 (2)	0.98045 (12)	0.0208 (5)	
O7	0.3028 (2)	0.6298 (2)	0.86878 (14)	0.0352 (7)	
OW1	0.3559 (2)	0.6465 (3)	0.67326 (16)	0.0298 (7)	
OW2	0.1725 (3)	0.5142 (4)	0.76325 (19)	0.0457 (9)	
OW3	0.2372 (2)	1.4313 (3)	0.64258 (18)	0.0349 (7)	
C1	0.0448 (2)	1.3358 (4)	0.5645 (2)	0.0235 (8)	
H1	0.0702	1.3998	0.5364	0.028*	
C2	−0.0140 (2)	1.3825 (4)	0.6028 (2)	0.0303 (9)	
H2	−0.0294	1.4770	0.6004	0.036*	
C3	−0.0502 (3)	1.2912 (4)	0.6446 (2)	0.0351 (10)	
H3	−0.0898	1.3218	0.6725	0.042*	
C4	−0.0276 (3)	1.1533 (4)	0.6451 (2)	0.0340 (10)	
H4	−0.0527	1.0873	0.6722	0.041*	
C5	0.0316 (2)	1.1135 (4)	0.60598 (18)	0.0219 (8)	
C6	0.0611 (2)	0.9704 (4)	0.6032 (2)	0.0227 (8)	
C7	0.0414 (3)	0.8645 (4)	0.6488 (2)	0.0314 (9)	
H7	0.0059	0.8809	0.6831	0.038*	
C8	0.0744 (3)	0.7355 (4)	0.6431 (2)	0.0381 (11)	
H8	0.0622	0.6621	0.6741	0.046*	
C9	0.1252 (3)	0.7130 (4)	0.5925 (2)	0.0319 (9)	
H9	0.1481	0.6244	0.5880	0.038*	
C10	0.1423 (2)	0.8217 (4)	0.5483 (2)	0.0253 (8)	
H10	0.1764	0.8061	0.5126	0.030*	
C11	0.4853 (2)	0.7458 (4)	0.5792 (2)	0.0246 (8)	
H11	0.4533	0.6805	0.5458	0.030*	
C12	0.5594 (3)	0.7043 (4)	0.6264 (2)	0.0313 (9)	
H12	0.5795	0.6131	0.6234	0.038*	
C13	0.6037 (3)	0.7976 (4)	0.6779 (2)	0.0334 (10)	
H13	0.6527	0.7695	0.7129	0.040*	
C14	0.5763 (2)	0.9309 (4)	0.6779 (2)	0.0278 (9)	
H14	0.6066	0.9970	0.7121	0.033*	
C15	0.5031 (2)	0.9676 (3)	0.62682 (19)	0.0190 (7)	
C16	0.4712 (2)	1.1104 (3)	0.61762 (19)	0.0197 (8)	
C17	0.5086 (2)	1.2213 (4)	0.6617 (2)	0.0272 (9)	
H17	0.5558	1.2073	0.7019	0.033*	
C18	0.4759 (3)	1.3511 (4)	0.6460 (2)	0.0294 (9)	
H18	0.4998	1.4281	0.6755	0.035*	
C19	0.4070 (2)	1.3679 (4)	0.5859 (2)	0.0279 (9)	
H19	0.3850	1.4572	0.5724	0.033*	
C20	0.3711 (2)	1.2534 (4)	0.5464 (2)	0.0229 (8)	
H20	0.3229	1.2645	0.5069	0.027*	
C21	0.2685 (2)	1.0230 (3)	0.66996 (17)	0.0139 (7)	
C22	0.2545 (2)	1.0030 (3)	0.75023 (17)	0.0144 (7)	
C23	0.2148 (2)	1.1040 (3)	0.78405 (17)	0.0147 (7)	
H23	0.1922	1.1824	0.7551	0.018*	
C24	0.2075 (2)	1.0922 (3)	0.85994 (17)	0.0140 (7)	
C25	0.2373 (2)	0.9754 (3)	0.90225 (18)	0.0151 (7)	
H25	0.2345	0.9684	0.9545	0.018*	
C26	0.2715 (2)	0.8687 (3)	0.86660 (18)	0.0156 (7)	
C27	0.2982 (2)	0.7339 (3)	0.90773 (19)	0.0194 (8)	
C28	0.2803 (2)	0.8831 (3)	0.79174 (18)	0.0155 (7)	
H28	0.3043	0.8103	0.7683	0.019*	
C29	0.1689 (2)	1.2103 (3)	0.89620 (18)	0.0164 (7)	
H1W1	0.319 (3)	0.597 (4)	0.665 (2)	0.023 (13)*	
H2W1	0.331 (3)	0.719 (5)	0.656 (2)	0.048 (15)*	
H1W3	0.247 (4)	1.359 (5)	0.646 (3)	0.06 (2)*	
H2W3	0.210 (2)	1.441 (4)	0.679 (2)	0.025 (11)*	
H1W2	0.167 (3)	0.445 (5)	0.785 (3)	0.040 (14)*	
H2W2	0.213 (4)	0.554 (6)	0.786 (3)	0.07 (2)*	
HO2	0.230 (3)	1.033 (4)	0.42861 (17)	0.046 (13)*	

### Atomic displacement parameters (Å^2^)

**Table:**

	*U*^11^	*U*^22^	*U*^33^	*U*^12^	*U*^13^	*U*^23^
Cu1	0.0200 (2)	0.0156 (2)	0.0150 (2)	−0.00017 (19)	0.00539 (16)	0.00193 (16)
Cu2	0.0206 (2)	0.0145 (2)	0.0144 (2)	0.00010 (19)	0.00470 (16)	−0.00173 (16)
N1	0.0169 (16)	0.0198 (16)	0.0206 (15)	0.0001 (13)	0.0025 (12)	0.0030 (12)
N2	0.0188 (17)	0.0179 (15)	0.0238 (15)	−0.0013 (13)	0.0020 (12)	0.0047 (12)
N3	0.0219 (17)	0.0127 (14)	0.0219 (15)	0.0010 (13)	0.0074 (12)	0.0013 (12)
N4	0.0194 (16)	0.0145 (14)	0.0212 (15)	−0.0003 (13)	0.0089 (12)	−0.0005 (12)
O1	0.0297 (16)	0.0224 (13)	0.0139 (12)	−0.0009 (11)	0.0103 (10)	−0.0014 (10)
O2	0.0231 (14)	0.0219 (13)	0.0126 (12)	0.0059 (11)	0.0037 (10)	0.0001 (10)
O3	0.0273 (14)	0.0140 (12)	0.0129 (11)	0.0038 (11)	0.0055 (10)	0.0039 (9)
O4	0.0269 (15)	0.0121 (12)	0.0143 (11)	0.0023 (10)	0.0073 (10)	−0.0004 (9)
O5	0.0297 (16)	0.0229 (13)	0.0206 (13)	0.0099 (12)	0.0034 (11)	−0.0018 (10)
O6	0.0289 (15)	0.0148 (12)	0.0171 (12)	−0.0018 (11)	0.0014 (10)	0.0057 (9)
O7	0.073 (2)	0.0154 (13)	0.0258 (14)	0.0123 (14)	0.0284 (14)	0.0069 (11)
OW1	0.0338 (19)	0.0219 (16)	0.0319 (16)	0.0027 (15)	0.0034 (13)	0.0036 (13)
OW2	0.072 (3)	0.036 (2)	0.0321 (18)	0.017 (2)	0.0165 (19)	0.0101 (16)
OW3	0.045 (2)	0.0226 (17)	0.0410 (18)	0.0011 (15)	0.0173 (15)	−0.0035 (14)
C1	0.023 (2)	0.023 (2)	0.0257 (19)	0.0012 (16)	0.0068 (15)	0.0026 (15)
C2	0.030 (2)	0.031 (2)	0.031 (2)	0.0102 (19)	0.0102 (17)	−0.0026 (17)
C3	0.027 (2)	0.053 (3)	0.029 (2)	0.015 (2)	0.0134 (17)	0.0054 (19)
C4	0.025 (2)	0.045 (3)	0.034 (2)	0.002 (2)	0.0132 (18)	0.0188 (19)
C5	0.017 (2)	0.031 (2)	0.0164 (17)	−0.0006 (16)	0.0000 (14)	0.0075 (15)
C6	0.017 (2)	0.024 (2)	0.0252 (19)	−0.0016 (16)	0.0003 (15)	0.0075 (15)
C7	0.027 (2)	0.035 (2)	0.032 (2)	−0.0047 (19)	0.0059 (17)	0.0130 (18)
C8	0.032 (3)	0.030 (2)	0.047 (3)	−0.010 (2)	−0.003 (2)	0.0211 (19)
C9	0.027 (2)	0.019 (2)	0.044 (2)	−0.0035 (17)	−0.0032 (18)	0.0053 (17)
C10	0.021 (2)	0.0183 (19)	0.033 (2)	−0.0046 (16)	−0.0033 (16)	0.0037 (15)
C11	0.023 (2)	0.0162 (18)	0.037 (2)	0.0001 (16)	0.0105 (16)	−0.0020 (15)
C12	0.036 (3)	0.020 (2)	0.042 (2)	0.0066 (19)	0.0158 (19)	0.0030 (17)
C13	0.020 (2)	0.033 (2)	0.045 (2)	0.0023 (19)	0.0013 (17)	0.0094 (19)
C14	0.023 (2)	0.029 (2)	0.029 (2)	−0.0034 (18)	0.0017 (16)	0.0028 (16)
C15	0.018 (2)	0.0194 (18)	0.0217 (18)	−0.0025 (15)	0.0086 (14)	−0.0011 (14)
C16	0.018 (2)	0.0201 (18)	0.0233 (18)	−0.0016 (16)	0.0092 (15)	−0.0012 (14)
C17	0.025 (2)	0.026 (2)	0.030 (2)	−0.0043 (17)	0.0042 (16)	−0.0067 (16)
C18	0.033 (2)	0.023 (2)	0.034 (2)	−0.0073 (18)	0.0109 (18)	−0.0060 (16)
C19	0.029 (2)	0.0156 (19)	0.044 (2)	−0.0005 (17)	0.0174 (18)	0.0015 (17)
C20	0.024 (2)	0.0190 (18)	0.0282 (19)	0.0001 (16)	0.0112 (15)	0.0012 (15)
C21	0.0168 (18)	0.0121 (16)	0.0116 (15)	−0.0035 (14)	0.0005 (13)	−0.0018 (12)
C22	0.0155 (19)	0.0145 (16)	0.0133 (16)	−0.0006 (14)	0.0035 (13)	−0.0012 (13)
C23	0.0167 (18)	0.0128 (16)	0.0129 (16)	−0.0013 (14)	−0.0005 (13)	0.0010 (12)
C24	0.0152 (18)	0.0133 (16)	0.0142 (16)	0.0002 (13)	0.0050 (13)	−0.0027 (12)
C25	0.0182 (19)	0.0152 (17)	0.0123 (15)	−0.0033 (14)	0.0045 (13)	0.0010 (12)
C26	0.0146 (18)	0.0143 (17)	0.0175 (16)	−0.0002 (14)	0.0024 (13)	0.0006 (13)
C27	0.019 (2)	0.0190 (18)	0.0234 (19)	0.0050 (15)	0.0113 (15)	0.0090 (14)
C28	0.0168 (18)	0.0148 (16)	0.0158 (16)	0.0001 (15)	0.0053 (13)	−0.0003 (13)
C29	0.0131 (18)	0.0173 (18)	0.0183 (17)	−0.0004 (14)	0.0021 (13)	−0.0071 (14)

### Geometric parameters (Å, º)

**Table:**

Cu1—O2	1.903 (2)	C3—H3	0.9500
Cu1—O1^i^	1.961 (2)	C4—C5	1.373 (5)
Cu1—N1	2.015 (3)	C4—H4	0.9500
Cu1—N2	2.026 (3)	C5—C6	1.476 (5)
Cu1—O3	2.305 (2)	C6—C7	1.393 (5)
Cu1—O5^i^	2.935 (2)	C7—C8	1.377 (6)
Cu1—Cu2	3.5251 (6)	C7—H7	0.9500
Cu2—O2	1.918 (2)	C8—C9	1.377 (6)
Cu2—O6^ii^	1.980 (2)	C8—H8	0.9500
Cu2—N3	2.017 (3)	C9—C10	1.383 (5)
Cu2—N4	2.019 (3)	C9—H9	0.9500
Cu2—O4	2.301 (2)	C10—H10	0.9500
Cu2—O7^ii^	2.866 (2)	C11—C12	1.388 (5)
N1—C1	1.332 (4)	C11—H11	0.9500
N1—C5	1.348 (4)	C12—C13	1.382 (5)
N2—C10	1.340 (4)	C12—H12	0.9500
N2—C6	1.353 (5)	C13—C14	1.369 (5)
N3—C11	1.337 (4)	C13—H13	0.9500
N3—C15	1.343 (4)	C14—C15	1.396 (5)
N4—C16	1.337 (4)	C14—H14	0.9500
N4—C20	1.348 (4)	C15—C16	1.479 (5)
O1—C29	1.263 (4)	C16—C17	1.397 (5)
O1—Cu1^iii^	1.961 (2)	C17—C18	1.375 (5)
O2—HO2	0.90 (2)	C17—H17	0.9500
O3—C21	1.263 (4)	C18—C19	1.396 (6)
O4—C21	1.261 (4)	C18—H18	0.9500
O5—C29	1.242 (4)	C19—C20	1.380 (5)
O6—C27	1.272 (4)	C19—H19	0.9500
O6—Cu2^iv^	1.980 (2)	C20—H20	0.9500
O7—C27	1.239 (4)	C21—C22	1.517 (4)
OW1—H1W1	0.76 (4)	C22—C23	1.389 (4)
OW1—H2W1	0.84 (5)	C22—C28	1.396 (4)
OW2—H1W2	0.79 (4)	C23—C24	1.395 (4)
OW2—H2W2	0.79 (6)	C23—H23	0.9500
OW3—H1W3	0.72 (5)	C24—C25	1.393 (4)
OW3—H2W3	0.89 (4)	C24—C29	1.522 (4)
C1—C2	1.380 (5)	C25—C26	1.397 (4)
C1—H1	0.9500	C25—H25	0.9500
C2—C3	1.376 (5)	C26—C28	1.386 (4)
C2—H2	0.9500	C26—C27	1.518 (4)
C3—C4	1.389 (6)	C28—H28	0.9500
			
O2—Cu1—O1^i^	94.05 (10)	N1—C5—C4	122.1 (3)
O2—Cu1—N1	173.34 (10)	N1—C5—C6	114.1 (3)
O1^i^—Cu1—N1	92.36 (10)	C4—C5—C6	123.9 (3)
O2—Cu1—N2	93.86 (11)	N2—C6—C7	121.2 (3)
O1^i^—Cu1—N2	165.70 (11)	N2—C6—C5	115.1 (3)
N1—Cu1—N2	80.24 (12)	C7—C6—C5	123.7 (3)
O2—Cu1—O3	89.55 (9)	C8—C7—C6	118.8 (4)
O1^i^—Cu1—O3	106.08 (9)	C8—C7—H7	120.6
N1—Cu1—O3	86.97 (10)	C6—C7—H7	120.6
N2—Cu1—O3	85.88 (10)	C7—C8—C9	119.9 (4)
O2—Cu1—O5^i^	71.33 (8)	C7—C8—H8	120.1
O1^i^—Cu1—O5^i^	49.27 (8)	C9—C8—H8	120.1
N1—Cu1—O5^i^	114.46 (9)	C8—C9—C10	118.9 (4)
N2—Cu1—O5^i^	123.06 (9)	C8—C9—H9	120.5
O3—Cu1—O5^i^	145.28 (8)	C10—C9—H9	120.5
O2—Cu1—Cu2	22.78 (7)	N2—C10—C9	121.8 (4)
O1^i^—Cu1—Cu2	104.47 (7)	N2—C10—H10	119.1
N1—Cu1—Cu2	152.20 (8)	C9—C10—H10	119.1
N2—Cu1—Cu2	87.22 (9)	N3—C11—C12	121.7 (3)
O3—Cu1—Cu2	67.34 (6)	N3—C11—H11	119.1
O5^i^—Cu1—Cu2	93.18 (5)	C12—C11—H11	119.1
O2—Cu2—O6^ii^	93.88 (10)	C13—C12—C11	119.0 (3)
O2—Cu2—N3	174.49 (11)	C13—C12—H12	120.5
O6^ii^—Cu2—N3	91.55 (10)	C11—C12—H12	120.5
O2—Cu2—N4	94.59 (11)	C14—C13—C12	119.5 (4)
O6^ii^—Cu2—N4	165.79 (11)	C14—C13—H13	120.3
N3—Cu2—N4	79.91 (11)	C12—C13—H13	120.3
O2—Cu2—O4	91.51 (9)	C13—C14—C15	118.6 (4)
O6^ii^—Cu2—O4	101.62 (9)	C13—C14—H14	120.7
N3—Cu2—O4	88.26 (10)	C15—C14—H14	120.7
N4—Cu2—O4	89.54 (9)	N3—C15—C14	122.0 (3)
O2—Cu2—O7^ii^	71.02 (9)	N3—C15—C16	114.7 (3)
O6^ii^—Cu2—O7^ii^	50.63 (8)	C14—C15—C16	123.3 (3)
N3—Cu2—O7^ii^	112.05 (10)	N4—C16—C17	121.8 (3)
N4—Cu2—O7^ii^	122.32 (9)	N4—C16—C15	114.4 (3)
O4—Cu2—O7^ii^	143.84 (8)	C17—C16—C15	123.8 (3)
O2—Cu2—Cu1	22.60 (7)	C18—C17—C16	119.0 (4)
O6^ii^—Cu2—Cu1	105.09 (7)	C18—C17—H17	120.5
N3—Cu2—Cu1	154.86 (8)	C16—C17—H17	120.5
N4—Cu2—Cu1	86.86 (8)	C17—C18—C19	118.8 (4)
O4—Cu2—Cu1	70.23 (6)	C17—C18—H18	120.6
O7^ii^—Cu2—Cu1	93.09 (6)	C19—C18—H18	120.6
C1—N1—C5	118.8 (3)	C20—C19—C18	119.3 (3)
C1—N1—Cu1	125.9 (2)	C20—C19—H19	120.3
C5—N1—Cu1	115.2 (2)	C18—C19—H19	120.3
C10—N2—C6	119.4 (3)	N4—C20—C19	121.5 (3)
C10—N2—Cu1	125.3 (2)	N4—C20—H20	119.2
C6—N2—Cu1	113.5 (2)	C19—C20—H20	119.2
C11—N3—C15	119.0 (3)	O4—C21—O3	125.5 (3)
C11—N3—Cu2	127.0 (2)	O4—C21—C22	117.8 (3)
C15—N3—Cu2	113.4 (2)	O3—C21—C22	116.6 (3)
C16—N4—C20	119.4 (3)	C23—C22—C28	118.3 (3)
C16—N4—Cu2	113.6 (2)	C23—C22—C21	120.3 (3)
C20—N4—Cu2	125.9 (2)	C28—C22—C21	121.3 (3)
C29—O1—Cu1^iii^	114.7 (2)	C22—C23—C24	121.0 (3)
Cu1—O2—Cu2	134.61 (12)	C22—C23—H23	119.5
Cu1—O2—HO2	110 (3)	C24—C23—H23	119.5
Cu2—O2—HO2	115 (3)	C25—C24—C23	120.2 (3)
C21—O3—Cu1	118.31 (19)	C25—C24—C29	120.8 (3)
C21—O4—Cu2	123.51 (19)	C23—C24—C29	119.0 (3)
C27—O6—Cu2^iv^	113.2 (2)	C24—C25—C26	119.0 (3)
H1W1—OW1—H2W1	98 (4)	C24—C25—H25	120.5
H1W2—OW2—H2W2	109 (5)	C26—C25—H25	120.5
H1W3—OW3—H2W3	101 (5)	C28—C26—C25	120.2 (3)
N1—C1—C2	122.0 (3)	C28—C26—C27	118.5 (3)
N1—C1—H1	119.0	C25—C26—C27	121.3 (3)
C2—C1—H1	119.0	O7—C27—O6	124.4 (3)
C3—C2—C1	119.5 (4)	O7—C27—C26	118.4 (3)
C3—C2—H2	120.2	O6—C27—C26	117.2 (3)
C1—C2—H2	120.2	C26—C28—C22	121.0 (3)
C2—C3—C4	118.4 (4)	C26—C28—H28	119.5
C2—C3—H3	120.8	C22—C28—H28	119.5
C4—C3—H3	120.8	O5—C29—O1	125.3 (3)
C5—C4—C3	119.2 (4)	O5—C29—C24	118.2 (3)
C5—C4—H4	120.4	O1—C29—C24	116.5 (3)
C3—C4—H4	120.4		
			
C5—N1—C1—C2	−0.6 (5)	C14—C15—C16—N4	176.3 (3)
Cu1—N1—C1—C2	−176.2 (3)	N3—C15—C16—C17	179.3 (3)
N1—C1—C2—C3	1.0 (6)	C14—C15—C16—C17	−3.5 (5)
C1—C2—C3—C4	−1.6 (6)	N4—C16—C17—C18	−2.7 (5)
C2—C3—C4—C5	1.9 (6)	C15—C16—C17—C18	177.1 (3)
C1—N1—C5—C4	0.8 (5)	C16—C17—C18—C19	−0.6 (5)
Cu1—N1—C5—C4	176.9 (3)	C17—C18—C19—C20	3.1 (6)
C1—N1—C5—C6	−179.9 (3)	C16—N4—C20—C19	−0.9 (5)
Cu1—N1—C5—C6	−3.8 (4)	Cu2—N4—C20—C19	166.0 (3)
C3—C4—C5—N1	−1.5 (6)	C18—C19—C20—N4	−2.5 (5)
C3—C4—C5—C6	179.3 (4)	Cu2—O4—C21—O3	6.5 (5)
C10—N2—C6—C7	1.7 (5)	Cu2—O4—C21—C22	−173.1 (2)
Cu1—N2—C6—C7	−163.8 (3)	Cu1—O3—C21—O4	53.4 (4)
C10—N2—C6—C5	179.6 (3)	Cu1—O3—C21—C22	−127.0 (2)
Cu1—N2—C6—C5	14.2 (4)	O4—C21—C22—C23	−180.0 (3)
N1—C5—C6—N2	−7.0 (4)	O3—C21—C22—C23	0.4 (5)
C4—C5—C6—N2	172.3 (4)	O4—C21—C22—C28	−0.5 (5)
N1—C5—C6—C7	170.9 (3)	O3—C21—C22—C28	179.9 (3)
C4—C5—C6—C7	−9.8 (6)	C28—C22—C23—C24	5.8 (5)
N2—C6—C7—C8	−0.3 (6)	C21—C22—C23—C24	−174.7 (3)
C5—C6—C7—C8	−178.1 (3)	C22—C23—C24—C25	−2.5 (5)
C6—C7—C8—C9	−0.6 (6)	C22—C23—C24—C29	175.6 (3)
C7—C8—C9—C10	0.2 (6)	C23—C24—C25—C26	−2.5 (5)
C6—N2—C10—C9	−2.1 (5)	C29—C24—C25—C26	179.4 (3)
Cu1—N2—C10—C9	161.5 (3)	C24—C25—C26—C28	4.2 (5)
C8—C9—C10—N2	1.1 (6)	C24—C25—C26—C27	−174.6 (3)
C15—N3—C11—C12	−0.1 (5)	Cu2^iv^—O6—C27—O7	0.6 (5)
Cu2—N3—C11—C12	−170.4 (3)	Cu2^iv^—O6—C27—C26	179.1 (2)
N3—C11—C12—C13	3.5 (6)	C28—C26—C27—O7	−21.4 (5)
C11—C12—C13—C14	−4.1 (6)	C25—C26—C27—O7	157.4 (3)
C12—C13—C14—C15	1.5 (6)	C28—C26—C27—O6	160.0 (3)
C11—N3—C15—C14	−2.6 (5)	C25—C26—C27—O6	−21.2 (5)
Cu2—N3—C15—C14	168.9 (3)	C25—C26—C28—C22	−0.8 (5)
C11—N3—C15—C16	174.6 (3)	C27—C26—C28—C22	178.0 (3)
Cu2—N3—C15—C16	−13.9 (4)	C23—C22—C28—C26	−4.1 (5)
C13—C14—C15—N3	2.0 (5)	C21—C22—C28—C26	176.4 (3)
C13—C14—C15—C16	−175.0 (3)	Cu1^iii^—O1—C29—O5	−18.6 (4)
C20—N4—C16—C17	3.5 (5)	Cu1^iii^—O1—C29—C24	160.4 (2)
Cu2—N4—C16—C17	−164.9 (3)	C25—C24—C29—O5	−175.9 (3)
C20—N4—C16—C15	−176.3 (3)	C23—C24—C29—O5	6.0 (5)
Cu2—N4—C16—C15	15.3 (3)	C25—C24—C29—O1	5.1 (5)
N3—C15—C16—N4	−0.9 (4)	C23—C24—C29—O1	−173.0 (3)

Symmetry codes: (i) *x*, −*y*+5/2, *z*−1/2; (ii) *x*, −*y*+3/2, *z*−1/2; (iii) *x*, −*y*+5/2, *z*+1/2; (iv) *x*, −*y*+3/2, *z*+1/2.

### Hydrogen-bond geometry (Å, º)

**Table:**

*D*—H···*A*	*D*—H	H···*A*	*D*···*A*	*D*—H···*A*
O*W*1—H1*W*1···O*W*3^v^	0.76 (4)	2.09 (4)	2.834 (5)	168 (4)
O*W*1—H2*W*1···O4	0.84 (5)	2.08 (5)	2.878 (4)	159 (4)
O*W*3—H1*W*3···O3	0.72 (5)	2.18 (5)	2.880 (4)	165 (6)
O*W*3—H2*W*3···O*W*2^vi^	0.89 (4)	1.88 (4)	2.729 (5)	161 (4)
O*W*2—H1*W*2···O5^v^	0.79 (4)	1.94 (5)	2.716 (5)	164 (4)
O*W*2—H2*W*2···O7	0.79 (6)	2.01 (6)	2.774 (6)	163 (6)
O2—H*O*2···O5^i^	0.90 (2)	2.35 (3)	2.943 (3)	123 (3)
O2—H*O*2···O7^ii^	0.90 (2)	2.37 (4)	2.884 (3)	116 (3)

Symmetry codes: (i) *x*, −*y*+5/2, *z*−1/2; (ii) *x*, −*y*+3/2, *z*−1/2; (v) *x*, *y*−1, *z*; (vi) *x*, *y*+1, *z*.

### *M*···*M* separation (Å) for some binuclear copper(II) complexes

bpy is 2,2'-bipyridine, OAc is acetate, phen is 1,10-phenanthroline, tmen is N,N,N,N-tetramethylenediamine and Fc is ferrocenyl.

**Table:**

Compound	Cu···Cu
(1)	3.5251 (6)
Cu_2_(µ-OH)(µ-H_2_O)(µ-OAc)(bpy)_2_](ClO_4_)_2_^a^	3.035 (2)
[Cu_2_(µ-OAc)_3_(bpy)_2_](ClO_4_)^a^	3.392 (1)
[Cu_2_(phen)_2_(µ-OH)(µ-OAc)](NO_3_)_2_.H_2_O^b^	3.017 (2)
[Cu_2_(phen)_2_(µ-OH)(µ-O_2_CEt)](NO_3_)_2_.H_2_O^b^	3.015 (2)

References: (a) Christou *et al.* (1990); (b) Tokii *et al.* (1992).
